# How is your mind-set? Proof of concept for the measurement of the level of emotional development

**DOI:** 10.1371/journal.pone.0215474

**Published:** 2019-04-18

**Authors:** Tanja Sappok, Julia Böhm, Joana Birkner, Gerhard Roth, Manuel Heinrich

**Affiliations:** 1 Ev. Krankenhaus Königin Elisabeth Herzberge, Behandlungszentrum für psychische Gesundheit bei Entwicklungsstörungen, Berlin, Germany; 2 Freie Universität Berlin, Fakultät für Erziehungswissenschaft und Psychologie, Berlin, Germany; 3 Universität Bremen, Fachbereich 2 Biologie, Neurobiologie, Institut für Hirnforschung, Bremen, Germany; 4 Freie Universität Berlin, Fakultät für Erziehungswissenschaft und Psychologie, AB Klinisch-Psychologische Intervention, Berlin, Germany; Univdersity Hospital of TübingenUniversitatsklinikum Tubingen, GERMANY

## Abstract

**Background:**

In persons with intellectual and developmental disabilities, not only cognitive brain functions, but also socio-emotional processing networks may be impaired. This study aims to validate the Scale of Emotional Development—Short (SED-S) to provide an instrument for the assessment of socio-emotional brain functions.

**Method:**

The SED-S was applied in 160 children aged 0–12 years. Criterion validity was investigated at item and scale level in terms of the agreement between the scale classification and the child’s chronological age. Additionally, interrater reliability and internal consistency were assessed.

**Results:**

For the majority of items, the expected response pattern emerged, showing the highest response probabilities in the respective target age groups. Agreement between the classification of the different SED-S domains and chronological age was high (*κ*_*w =*_ 0.95; exact agreement = 80.6%). Interrater reliability at domain level ranged from *κ*_*w*_ = .98 to 1.00 and internal consistency was high (α = .99).

**Conclusion:**

The study normed the SED-S in a sample of typically developing children and provides evidence for criterion validity on item, domain and scale level.

## Background

For a comprehensive understanding of mental functioning, the traditional focus on intellectual competencies has to be widened to acknowledge socio-emotional brain functions as well [1]. These socio-emotional competencies can be conceptualized according to the emergence of the respective social processing networks alongside the trajectory of typically developing infants [2]. Thus, this comprehensive view of mental functioning comprises various aspects such as object permanency, self-other-differentiation, secure bounding, stress regulation, affect differentiation, impulse control and theory of mind [3; 4]. The maturation of emotional brain functions is the product of various internal (epi-) genetic and sensory and external factors such as bounding experience, trauma, education and learning. In a bottom-up process, the neuronal substrate, i.e. the developing brain, modifies social interaction abilities, while in a top down-process, these interactive experiences shape the developing brain networks [5]. As such, emotional functioning occurs in coordination with various processes, including cognitive, sexual, motor and moral development [2]. Each developmental level is associated with specific emotional needs, motivations, and coping skills, which affects a person’s ability to adapt to the environment [6, 7]

Neuroanatomical, the ‘emotional brain’ is located in the various centres of the limbic system [8]. The *deep limbic level* includes the diencephalon (hypothalamus and brain stem, including periventricular grey and vegetative nuclei) and the central nucleus of the amygdala, and processes basal functions that are necessary for survival, such as feeding, reproduction, and fight-flight reactions. Moreover, autonomic functions and the stress-regulation system are located in this part of the brain. The processes are mostly unconscious and genetically/epigenetically determined, and are only minimally influenced by environmental factors [8]. These structures develop mainly prenatally and predominate during the first months of life, when sensory stimuli from within the body, such as bowel movements, and external stimuli, such as noise and touch, are integrated and processed. The ‘way of thinking’ in this early phase of life is triggered by the sensory input and is goal- and action-oriented [9].

The *mesolimbic system* starts to develop prenatally and further maturates within the first months and years of life. It consists of subcortical areas and comprises the basolateral amygdala, the ventral tegmental area and the nucleus accumbens/ventral striatum, which are both parts of the basal ganglia and also functions predominantly unconscious [8, 10, 11]. As a result of the sensory stimulation and integration during the first months of live, step by step object permanence arises at the end of the first year. Now the person has an inner picture of the outside environment, the first steps towards mentalization are taken [9]. However, in this stage, persons cannot discriminate between their own thoughts and the outside world: i.e., thinking is reality.

In the mesolimbic system, basic emotional functions such as anxiety, sadness, disgust, happiness and anger are determined. The emotional conditioning derives from the bonding experiences to the central attachment figures during early life: The developing child learns to acknowledge, differentiate, understand and regulate his or her own emotions through interactive contact with his or her caregiver [12]. Moreover, the nonverbal communication network, i.e. the recognition of emotional-communicative signals evolves. Stimulated by the constant interaction with the close carers, with around 2 years, the infant is able to differentiate between the self and the other [9]. Therefore, the person is able to distinguish between his or her own thoughts and the (outside) reality. This differentiation is practised and trained in role and imaginative plays, and thereby the pretend mode of thinking arises.

The brain areas of the mesolimbic system are also decisive for reward (endogenous opioids) and reward expectation (dopamine) as a basis for motivation [8].

The *upper limbic level* consists of the allocortical structures of the limbic system: that is, the prefrontal, orbitofrontal and ventromedial frontal cortex, the anterior cingulate cortex and the insular cortex [8]. The upper limbic system evolves in late childhood and adolescence and derives from socio-emotional experiences with the wider social environment, including friends, peers and more distant relatives. By the end of the forth year of life, the child is able to acknowledge that other persons have different feelings, thoughts, intentions, and motivations: The Theroy of Mind network developed and the child is able to mentalize. This is the prerequisite for more complex emotional states such as empathy, friendship, loyalty and moral thinking, which are all located in the upper limbic system. Socialization and social motivation develop via conscious emotional-social learning. Moreover, socio-emotional core competencies, such as impulse control, delayed gratification, frustration tolerance and balancing of the consequences of one’s own behaviour, are determined in this brain area [8]. Advantages and disadvantages of actions can be appreciated, risks assessed properly and behaviours planned accordingly. The social brain networks processing executive functions, risk assessment and reality awareness are situated in the upper limbic system.

According to ICD-11, disorders of intellectual development are a group of etiologically diverse conditions *originating during the developmental period* and characterized by significantly below average intellectual functioning and adaptive behavior that are approximately two or more standard deviations below the mean [13]. As such, not only cognitive brain functions as assessed with standardized intelligence tests, but also the socio-emotional competencies and the related brain networks as described above are impaired. Additional sensory impairments may further aggravate emotional reactivity and coping strategies [14]. Generally, individuals with intellectual and developmental disability (IDD) pass through the same stages as do typically developing children, but with an increased risk of delay and incompletion [15, 16]. The developmental approach may be particularly supportive in the treatment and care of persons with developmental delays as it gives insights in the respective brain networks active at a certain developmental level [4, 7, 17]. Persons with intellectual and developmental disability (IDD) show high point prevalence rates of mental disorders and challenging behaviours (41%) [18]. The recognition of the level of development of the socio-emotional brain circuits presents an additional perspective to the mechanisms leading to particular (problem) behaviours [19, 20]. Without a thorough maturation of the mesolimbic and upper limbic system, for example, one cannot expect impulse control and empathy, but rather external support for self-regulation and physical well-being. Insight into emotional functioning is therefore crucial for appropriate treatment and support in persons with IDD [21, 22]. Subsequently, better-tailored interventions and treatment options may arise, encourage the acquisition of functional life skills, and result in increased well-being.

The level of mental functioning comprising the socio-emotional competencies can be determined with the Scale of Emotional Development–Short (SED-S) which is based on the developmental approach proposed by Anton Došen [4, 23, 24]. This developmental model describes five levels of socio-emotional development, which are aligned to the milestones of the developmental trajectories during childhood and the therewith associated maturation of the respective brain circuits. The SED-S consists of 200 binary items and is applied in the form of a semi-structured interview with a parent or caregiver. It describes the five levels of emotional functioning depicted within eight domains [23–25]: Relating to his/her Own Body, Relating to Significant Others, Dealing with Change–Object Permanence, Differentiating Emotions, Relating to Peers, Engaging with the Material World, Communicating with Others, and Regulating Affect. With five binary items for each level of functioning in each of the eight domains, the SED-S results in a profile and an overall ED score. Being aware of the continuous nature of emotional development, the SED-S is conceptualized in a stage-wise model. This stage-wise approach seems beneficial when aiming to guide work in clinical practice and behavioural therapy, as it generates clear action-advice based on the assessed level of ED [4, 22].

This study aims to assess the psychometric properties of the SED-S. Therefore, the SED-S is applied in a sample of typically developing children aged 0 to 12 years. The current research is necessary to ensure that the behaviours described in the different items really reflect the actual target behaviours which can be observed at a certain reference age to support the criterion validity of the SED-S. In detail, (1) the age specificity of a certain behaviour for the respective developmental age-group, including the profile homogeneity, is used to assess criterion validity; and (2) the agreement between two different raters is used to assess interrater reliability.

## Method

### Setting and design

The study sample consists of 160 typically developing children aged 0 to 12 years who were recruited from 12/2016 to 06/2017 in different institutions such as kindergartens, schools, sports clubs, and day-care centres, as well as from the families of staff at the organizing institution. Interested parents were given comprehensive information about the study. Inclusion criteria for the study were: Child aged from 0 to 12 years, no developmental delay or mental illness, and a declaration of informed consent by the parents. Typical development was ensured by taking a thorough developmental and medical history with a parent or close carer about the child’s birth, developmental milestones and life events, and by reviewing the child’s personal health record (PCHR; “gelbes Untersuchungsheft”), which is taken regularly for all children in Germany from birth up to adolescence and completed by the primary physician. This personal child health record was thus available to be checked for every participating child. The SED-S was applied in semi-structured interview form by two psychologists experienced in the concept of ED. For the estimation of ED on the domain level, the domain with the highest number of items rated as applicable for a child was chosen. The overall level of ED was marked by the domain with the fourth-lowest score (Details c.f. [23, 24]) The assigned level of ED, as derived from the assessment with the SED-S, was compared with the child’s chronological age. Interrater reliability was assessed in 25 cases. For this purpose, the information provided by the parents was scored by two psychologists. One psychologist (rater 1) conducted the interview and the second psychologist (rater 2) attended this assessment. Both interviewers scored the provided information independently.

### Assessment of the level of ED

The scale was administered as a semi-structured interview by a trained psychologist familiar with the developmental approach for several years with one close carer (120 mothers, 24 fathers, 4 grandmothers, 10 caregivers from the Kindergarden, 2 others). The interview takes about one hour and refers to the child’s behavior within the past six weeks. On domain level, the stage with the highest number of items rated as ‘typical’ was assumed to provide the best estimation of the child’s level of ED. On scale level, a rank-based strategy is proposed, with the highest level of ED within the four lowest-ranking domains determining the overall result. Sappok et al. (2016), Morisse, Sappok, de Neve, and Dosen (2017) and Sappok et al. (2018) reported the administration and scoring of the SED-S in detail [23–25].

### Ethics

All parents provided informed consent. The procedures were in accordance with the ethical standards of the institutional ethics committees and with the 1964 Helsinki declaration and its later amendments. The study was approved by the Charité ethics committee, EA2/193/16 (approval granted 02/2017).

### Sample characteristics

The sample characteristics are displayed in Table 1. At least 30 children were included in each age group, and both genders were represented equally. Most children lived in their nuclear family and 65 lived with siblings (40.6%). In light of the different ages included, the majority of the children went to kindergarten, while one-third spent the day with a parent at home and a quarter went to school.

**Table 1:** Characteristics of the study sample.

		*Total (N = 160 = 100%)*	*Male*	*Age (in months)*	
		*n* (%)	*n* (%)	*M (SD)*	*Range*
Age group					
	0–6 months	30 (18.8)	19 (63.3)	3.5 (1.9)	0–6
	7–18 months	30 (18.8)	11 (36.7)	12.0 (3.5)	7–17
	19–36 months	30 (18.8)	15 (50)	26.6 (4.5)	20–35
	37–84 months	38 (23.8)	20 (52.6)	63.3 (13.2)	38–84
	85–156 months	32 (20)	14 (43.8)	119.9 (20)	85–150
Living situation					
	Nuclear family	134 (83.8)	66 (49.3)		
	Single parent family	12 (7.5)	4 (33.3)		
	Blended family	14 (8.8)	9 (64.3)		
Day care situation					
	At home (with a parent)	48 (30)	26 (54.2)		
	Kindergarten	72 (45)	35 (48.6)		
	School	40 (25)	18 (45)		

### Statistical analysis

All analyses were performed using *R*. There were no missing values on item scores.

1. Criterion Validity. At item level (1.1), agreement between the child’s chronological age and the behaviours described in the items of the respective SED-S level of ED was analysed. The proportion of children in a certain age group showing these behaviours was assessed. The items for a respective age group were used as reference and compared with the proportions of ‘yes’ responses in adjacent age groups. For the items constructed to assess SED-S level 1, the proportion of ‘yes’ answers in children from age group 1 was compared to the corresponding proportion for children in age group 2; for SED-S level 2, the results for children from age group 2 were compared to those in age groups 1 and 3, and so forth. These comparisons were based on the hypothesis that children in a certain age group will display the behaviours described in the items for the respective level of ED more frequently than children in adjacent age groups. Comparisons were made using Fisher’s exact test (one-sided). Items were considered inappropriate if a) the response probability was low (< 45%) in the target age group, or b) the response probability was not significantly higher in the reference age group when compared to adjacent age-groups (lack of age group specificity). Due to multiple testing, only differences with *p* < .01 were considered statistically significant.
  At the domain level and overall scale level (1.2), *a*greement of the child’s chronological age with the SED-S classification was determined in terms of quadratic weighted kappa (*κ*_w_) with bootstrapped 95% confidence levels (95% CI), as well as the percentage of exact agreement. Sex differences (1.3) were looked at by stratifying this analysis for both boys and girls. Moreover, median values on domain and overall scale level were compared for these two groups.
  Within-Profile Homogeneity (1.4) of the SED-S profiles was assessed using Cronbach’s alpha (α) as an overall index for internal consistency. In addition, the difference between the highest and lowest SED-S levels (min-max difference) was computed as an indicator for within-profile homogeneity. Since the sample consisted of typically developing infants who should have even SED-S profiles, internal consistency should be high and high min-max difference should be rare.
2. Interrater reliability Agreement between raters was determined in terms of percentage agreement and weighted kappa.

## Results

### Criterion validity at item level

For the majority of items, the response probability within the target age group was significantly higher compared to the adjacent age groups. For each item, Tables 2–9 display the proportions of ‘yes’ answers for every age group and the level of significance when comparing the target age group with the adjacent age groups. Appropriate items (response probability > 45%; age group specificity) are printed in bold.

**Table 2:** Response probability at item level; domain: Relating to his/her own body.

		Proportion of ‘yes’ responses per age group				
Content	Item	0–6 months	7–18 months	19–36 months	37–84 months	85–156 months
**Emotional states are largely determined by basic physical sensations and needs (hunger, thirst, pain, fatigue, cold)**	b1_1	.800	.200 ***	.033	.000	.000
**Only feels safe and secure in a familiar environment, i.e. when surrounded by** **familiar faces and stimuli (touch, smells, sounds, etc.)**	b1_2	.600	.133 ***	.000	.000	.000
**Passively enjoys sensory stimulation (e.g. being bathed or touched)**	b1_3	.933	.467 ***	.100	.000	.000
**Explores his/her body randomly by touching, grasping, (thumb) sucking etc.**	b1_4	.767	.100 ***	.000	.000	.000
Engages with his/her body by means of repetitive movements (flapping arms, rocking back and forth etc.) and vocalizations	b1_5	.433	.067 **	.033	.000	.000
**Uses mouth as well as hands to explore his/her environment**	b2_1	.533 **	.900	.300 ***	.000	.000
**Uses his/her body as an instrument to explore the immediate environment** **(e.g. switching the light on and off repeatedly etc.)**	b2_2	.267 ***	1.000	.500 ***	.000	.000
Smears feces and body fluids (saliva, blood, sperm)	b2_3	.133	.300	.133	.000	.000
**Uses his/her body to manipulate objects in the immediate environment**	k2_4	.433 ***	.933	.433 ***	.026	.000
Physical contact with caregivers (e.g. during bathing, brushing hair etc.) leads to pleasurable interactions	b2_5	.667 ***	1.000	.900	.079	.000
**Wants to do everything him-/herself (e.g. personal hygiene, meals, etc.)**	b3_1	.000	.533 ***	1.000	.447 ***	.031
**Seeks help from others or utilizes objects to overcome physical limitations** **(uses a chair to reach the cookie jar, for example)**	b3_2	.000	.500 ***	1.000	.474 ***	.125
**Tries to assert his/her will by means of provocative behavior (e.g. stripping off clothes, stomping, flinging him-/herself on the ground)**	b3_3	.000	.433 ***	1.000	.316 ***	.062
**Uses language (sometimes supported by gestures) to communicate**	b3_4	.000	.333 ***	1.000	.605 ***	.125
Goes to the toilet on his/her own, but needs help with hygiene	b3_5	.000	.033 **	.300	.395	.000
**Seeks role models of the same gender to identify with (e.g. police officers, firefighters, pop stars, caregivers etc.)**	b4_1	.000	.033	.433 ***	1.000	.656 ***
**Has developed a gender identity**	b4_2	.000	.033	.367 ***	1.000	.750 ***
Shows a sense of shame/modesty (closes the door when using the toilet, for example)	b4_3	.000	.033	.133 ***	.658	.688
**Seeks to imitate role models in terms of appearance (e.g. by adopting hair and clothing styles, necklaces, bracelets etc.)**	b4_4	.000	.033	.267 ***	.868	.500 ***
**Wants to choose clothing according to personal taste, regardless of the weather or occasion**	b4_5	.000	.033	.233 ***	.632	.156 ***
**Seeks to assert his/her position in the peer group by means of his/her appearance**	b5_1	.000	.033	.067	.579 **	.875
Is eager to demonstrate physical prowess and compete with others	b5_2	.000	.033	.133	.711	.875
**Shows modesty/a sense of shame in respect to sexuality**	b5_3	.000	.033	.000	.474 ***	.875
**Is able to assess his/her physical abilities accurately**	b5_4	.000	.033	.067	.684 **	.969
**Is concerned with his/her appearance and increasingly able to gauge** **how he/she is perceived by others realistically**	b5_5	.000	.033	.000	.474 ***	.969

*Note*. *N* = 160. Response probabilities of relevant age groups are highlighted in grey. The reference age group is shaded.

* *p* < .05.

** *p* < .01.

*** *p* < .001.

Appropriate items (response probability > 45%; age group specificity) are printed in bold letters.

**Table 3:** Response probability at item level; domain: Relating to Significant Others.

		Proportion of ‘yes’ responses per age group				
Content	Item	0–6 months	7–18 months	19–36 months	37–84 months	85–156 months
**Social interaction mainly arises when basic needs (e.g. food, hygiene, touch) are being met**	s1_1	.800	.067 ***	.000	.000	.000
**Is soothed by physical contact with emotionally significant others (hugging, rocking etc.)**	s1_2	.933	.433 ***	.133	.000	.000
**Primarily interacts with his/her environment via the proximal senses (touch, smell and taste)**	s2_3	.467	.033 ***	.000	.000	.000
**Enjoys feeling the spatial boundaries of his/her body through intensive physical contact**	s1_4	1.000	.467 ***	.167	.000	.000
Contact is more difficult when there is a lot of commotion or noise (sensory overload)	s1_5	.533	.267 *	.067	.000	.000
Sense of wellbeing is dependent on the presence of emotionally significant others	s2_1	.800	.933	.567 **	.053	.031
**Shows a preference for contact with emotionally significant others**	s2_2	.633 ***	1.000	.767 **	.132	.000
Stays close to emotionally significant others in unfamiliar surroundings	s2_3	.200 ***	.867	.600 *	.105	.000
Protests when contact with emotionally significant others is lost or ended	s2_4	.400 *	.700	.333 **	.026	.031
**Actively seeks physical proximity to emotionally significant others and follows them like a shadow**	s2_5	.100 ***	.633	.133 ***	.026	.031
**Insists on getting his/her way**	s3_1	.000	.300 ***	.933	.447 ***	.094
**Wants attention and tests limits**	s3_2	.000	.433 ***	.967	.526 ***	.062
**Is open to alternative suggestions**	s3_3	.000	.333 ***	.933	.553 ***	.156
Only obeys rules when authority figures are present	s3_4	.000	.200 *	.433	.026 ***	.000
**Persistently opposes others' requests ("No!") in order to assert his/her will**	s3_5	.000	.233 ***	.900	.263 ***	.062
Solicits the opinion and seeks the approval of emotionally significant others	s4_1	.000	.000	.433 ***	.842	.625 *
**Wants to assume the role of authority figures ("deputy sheriff")**	s4_2	.000	.000	.300 ***	.816	.438 ***
Makes decisions on his/her own and is aware of the immediate consequences (when crossing the street, for example)	s4_3	.000	.000	.167 ***	.868	.750
**Identifies with role models and attempts to emulate their characteristic behavior**	s4_4	.000	.000	.333 ***	.895	.625 **
Wants to be perceived and treated in accordance with his/her gender	s4_5	.000	.000	.100 ***	.868	.719
Is eager to compete with emotionally significant others	s5_1	.000	.000	.067	.816	.906
**Conforms to social norms and rules even when no authority figures are present**	s5_2	.000	.000	.100	.763 **	1.000
Models behavior on peers more than on authority figures	s5_3	.000	.000	.033	.368 *	.594
**Looks to individuals outside his/her immediate everyday environment (e.g. athletic coaches etc.) for guidance**	s5_4	.000	.000	.000	.421 ***	.844
**Seeks to gain approval by taking on responsibilities or tasks and pointing accomplishments out to emotionally significant others ("look what I did")**	s5_5	.000	.000	.067	.711 **	.969

*Note*. *N* = 160. Response probabilities of relevant age groups are highlighted in grey. The reference age group is shaded.

* *p* < .05.

** *p* < .01.

*** *p* < .001.

Appropriate items (response probability > 45%; age group specificity) are printed in bold letters.

**Table 4:** Response probability at item level; domain: Dealing with change / object permanence.

		Proportion of ‘yes’ responses per age group				
Content	Item	0–6 months	7–18 months	19–36 months	37–84 months	85–156 months
**Emotional states are primarily determined by the immediate situation**	o1_1	.867	.267 ***	.000	.000	.000
**Emotional states are primarily determined by the immediate situation**	o1_2	.833	.100 ***	.000	.000	.000
**Persons or objects that cannot be perceived using any sense no longer exist**	o1_3	.767	.000 ***	.000	.000	.000
**Does not look for hidden/lost objects ("out of sight, out of mind")**	o1_4	.800	.067 ***	.000	.000	.000
**Lives entirely in the "here and now"**	o1_5	.967	.433 ***	.000	.000	.000
**Is tense and upset when separated from significant others**	o2_1	.167 ***	.733	.267 ***	.000	.000
**Significant others provide a sense of security in unfamiliar situations**	o2_2	.667 ***	1.000	.667 ***	.053	.000
**Briefly looks for things that have disappeared from sight**	o2_3	.233 ***	.933	.367 ***	.026	.031
Enjoys playing peek-a-boo and hide-and-seek	o2_4	.433 ***	.900	.667 *	.053	.000
Frequently seeks verbal reassurance from significant others	o2_5	.167 ***	.633	.700	.053	.000
Is upset at loss of transitional objects (i.e. emotionally charged "comfort" objects)	o3_1	.067	.500	.600	.184 ***	.062
Transitional/comfort objects provide a sense of security	o3_2	.033	.533	.633	.447	.062
**Specifically looks for things and people that can no longer be seen/heard**	o3_3	.000	.400 ***	.967	.421 ***	.094
**Emotional bonds are maintained over a distance (e.g. over the telephone)**	o3_4	.000	.267 ***	.967	.605 ***	.125
Shows resentment after being separated from significant others for longer periods of time	o3_5	.000	.067	.133	.132	.031
Can manage transitions between familiar social contexts (e.g. residential and work settings) alone	o4_1	.000	.100	1.000	1.000	.688 ***
Is able to part with comfort objects in familiar environments	o4_2	.000	.067	.700	.737	.344 ***
Authority figures provide reassurance in unfamiliar situations	o4_3	.000	.100	1.000	.974	.531 ***
Can engage in familiar activities in unfamiliar environments	o4_4	.000	.067	.867 *	1.000	.594 ***
**Is insecure in unfamiliar environments when no authority figures are present**	o4_5	.000	.033	.833 **	.553	.250 **
Initiates social activities on his/her own	o5_1	.000	.033	.367	.868 *	1.000
Explores unfamiliar environments of his/her own accord	o5_2	.000	.033	.167	.763 *	.938
**Adapts to changing situational demands**	o5_3	.000	.033	.367	.737 ***	1.000
**Pursues interests beyond his/her familiar environment**	o5_4	.000	.033	.100	.684 ***	1.000
Applies familiar behavioral principles in new contexts	o5_5	.000	.033	.300	.842 *	1.000

*Note*. *N* = 160. Response probabilities of relevant age groups are highlighted in grey. The reference age group is shaded.

* *p* < .05.

** *p* < .01.

*** *p* < .001.

Appropriate items (response probability > 45%; age group specificity) are printed in bold letters.

**Table 5:** Response probability at item level; domain: Differentiating Emotions.

		Proportion of ‘yes’ responses per age group				
Content	Item	0–6 months	6–18 months	18–36 months	37–84 months	85–156 months
**Emotional states are expressed with the entire body**	e1_1	.900	.333 ***	.100	.000	.000
**The expression of emotions changes rapidly in response to stimuli Emotions are expressed intensely with no variation in degree**	e1_2	.900	.400 ***	.100	.000	.000
Emotions are expressed intensely with no variation in degree	e1_3	.267	.000 **	.000	.000	.000
Reacts to sensory overload by retreating or shutting out stimuli (e.g. covering his/her ears)	e1_4	.367	.067 **	.133	.000	.000
**Responds to aversive stimuli with a mix of tension, anxiety and anger**	e1_5	.733	.233 ***	.167	.000	.000
Reacts negatively when contact with significant others is not possible	e2_1	.633	.667	.333 **	.000	.031
Enjoys activities with significant others	e2_2	.800 *	1.000	.800 *	.132	.031
Dislikes being alone and seeks contact with (significant) others	e2_3	.333 ***	.867	.667	.053	.031
Emotional states can be influenced by attention from caregivers	e2_4	.700 ***	1.000	.800 *	.132	.031
Is noticeably tense in unfamiliar/confusing social situations	e2_5	.367	.600	.400	.000	.000
**Expresses anger when he/she doesn't get his/her way**	e3_1	.000	.567 ***	.967	.421 ***	.094
Shows defiance towards significant others	e3_2	.000	.333	.567	.237 **	.000
**Shows defiance towards significant others**	e3_3	.000	.300 ***	.967	.368 ***	.094
Is able to name own basic feelings (e.g. anger, sadness, fear, happiness)	e3_4	.000	.033 ***	.567	.579	.156
**Wants to be the center of attention**	e3_5	.000	.167 ***	.767	.289 ***	.000
Is afraid of making mistakes or doing something wrong	e4_1	.000	.000	.033 ***	.711	.500 *
**Is able to empathize with others (tries to console them, for example)**	e4_2	.000	.000	.733 **	.974	.750 **
**Shows feelings of guilt**	e4_3	.000	.000	.333 ***	.974	.719 **
Shows a sense of shame/modesty (facial expression)	e4_4	.000	.000	.367 ***	.895	.688 *
Is worried he/she won't be able to handle assigned tasks or responsibilities	e4_5	.000	.000	.000 ***	.632	.438
Is worried about not being accepted by peers	e5_1	.000	.000	.000	.553	.625
**Is concerned about his/her appearance**	e5_2	.000	.000	.000	.500 **	.844
**Strives to win the approval of peers**	e5_3	.000	.000	.033	.711 **	.969
**Is concerned about violating social conventions and rules**	e5_4	.000	.000	.000	.395 ***	.906
**Is able to reflect on and regulate his/her emotions**	e5_5	.000	.000	.033	.605 ***	.938

*Note*. *N* = 160. Response probabilities of relevant age groups are highlighted in grey. The reference age group is shaded.

* *p* < .05.

** *p* < .01.

*** *p* < .001.

Appropriate items (response probability > 45%; age group specificity) are printed in bold letters.

**Table 6:** Response probability at item level; domain: Relating to peers.

		Proportion of ‘yes’ responses per age group				
Content	Item	0–6 months	7–18 months	19–36 months	37–84 months	85–156 months
**Shows no interest in peers**	p1_1	.467	.000 ***	.000	.000	.000
Sees others as objects rather than beings who act and react	p1_2	.533	.233 *	.033	.000	.000
Interacts with peers according to fixed patterns	p2_3	.333	.167	.033	.000	.000
Actions elicit responses from peers	p1_4	.633	.400	.133	.000	.000
Forced contact with peers results in noticeable tension	p1_5	.067	.067	.067	.000	.000
**Engages in the same activities as peers, but independently of them** **(i.e. "parallel play"—playing side by side rather than with each other)**	p2_1	.267 ***	.933	.633 **	.000	.000
Is curious about peers and interacts with them briefly	p2_2	.400 ***	.867	.733	.053	.031
Tries to find out more about peers by watching, listening and/or touching	p2_3	.433 ***	.933	.667 *	.079	.031
Imitates peers when interacting with others	p2_4	.200 ***	.667	.700	.105	.031
Engages with peers when authority figures are present	p2_5	.200 ***	.867	.767	.079	.031
**Tries to impose his/her will on peers**	p3_1	.000	.367 ***	.900	.342 ***	.094
Is reluctant to share with peers	p3_2	.000	.433	.633	.079 ***	.000
Shows no regard for what peers want	p3_3	.000	.400 *	.700	.105 ***	.000
**Is jealous of peers**	p3_4	.000	.200 **	.567	.211 **	.031
Bosses peers around	p3_5	.000	.167 *	.400	.184 *	.000
Has regular, sustained contact with peers	p4_1	.000	.133	.967	1.000	.719 ***
**Makes friends with certain peers**	p4_2	.000	.000	.533 ***	.947	.688 **
**Imitates caregivers when interacting with peers**	p4_3	.000	.000	.367 **	.711	.250 ***
Frequently enjoys activities with peers for sustained periods, but is still primarily focused on what he/she wants	p4_4	.000	.033	.633	.789	.219 ***
Voluntarily shares with peers	p4_5	.000	.033	.367 ***	.921	.781
Maintains close friendships with peers	p5_1	.000	.000	.133	.711 *	.938
Is competitive with peers	p5_2	.000	.000	.100	.684 *	.875
Wants to "belong" and be popular with peers	p5_3	.000	.000	.067	.921	1.000
**Shows loyalty to friends**	p5_4	.000	.000	.000	.605 ***	1.000
**Consults with peers to find solutions to conflicts/problems**	p5_5	.000	.000	.067	.684 **	.969

*Note*. *N* = 160. Response probabilities of relevant age groups are highlighted in grey. The reference age group is shaded.

* *p* < .05. *

* *p* < .01.

*** *p* < .001.

Appropriate items (response probability > 45%; age group specificity) are printed in bold letters.

**Table 7:** Response probability at item level; domain: Engaging with the material world.

		Proportion of ‘yes’ responses per age group				
Content	Item	0–6 months	7–8 months	19–36 months	37–84 months	85–156 months
**Is mainly preoccupied with his/her own body**	m1_1	.633	.033 ***	.000	.000	.000
**Only engages with materials and objects within immediate reach**	m1_2	.700	.067 ***	.000	.000	.000
**Primarily engages in repetitive activities that stimulate the senses** **(e.g. rocking, making sounds, touching)**	m1_3	.733	.133 ***	.033	.000	.000
Enjoys handling and exploring amorphous materials such as water, sand or soap bubbles	m1_4	.733	.633	.067	.000	.000
**Explores things by feeling, licking and tasting them**	m1_5	.900	.500 ***	.033	.000	.000
**Manipulates objects repetitively for sustained periods of time (e.g. putting them into/taking them out of boxes, moving them back and forth, throwing them etc,)**	m2_1	.333 ***	.900	.400 ***	.000	.000
Interacting with significant others is the main focus of activities	m2_2	.300 *	.567	.333	.000	.000
**Enjoys interactions that involve repeatedly throwing, dropping and handing objects back and forth**	m2_3	.267 ***	.933	.400 ***	.026	.000
**Explores/examines materials and objects by kneading, hitting and shaking them**	m2_4	.267 ***	1.000	.633 ***	.000	.000
**Reaches for things he/she can see or hear**	m2_5	.467 ***	.967	.633 **	.000	.000
**Shares only reluctantly or at the request of significant others**	m3_1	.000	.267 **	.667	.132 ***	.000
**Engages in activities requiring fine motor skills, such as cutting and pasting**	m3_2	.000	.100 ***	.967	.500 ***	.125
**The activity itself is more important than the end result (has no interest in** **keeping drawings once they're finished, for example)**	m3_3	.000	.233 ***	.967	.158 ***	.000
Frequently examines and takes objects apart	m3_4	.000	.133 *	.367	.026 ***	.000
**Imitates others in play**	m3_5	.000	.233 ***	1.000	.447 ***	.031
Approaches peers during play and shared activities	m4_1	.000	.100	.933	.974	.719 **
**Likes games involving imagination (playing "pretend" and make-believe)**	m4_2	.000	.000	.600 ***	1.000	.562 ***
**Engages in creative activities such as painting and drawing**	m4_3	.000	.000	.367 ***	.974	.500 ***
**Shows imagination and creativity by varying colors, shapes etc. when working with materials**	m4_4	.000	.000	.233 ***	.868	.562 **
**Follows simple rules (like waiting his/her turn) when playing games**	m4_5	.000	.000	.367 ***	.947	.594 ***
Can share materials, tasks and responsibilities in activities with peers	m5_1	.000	.000	.300	.895	1.000
The primary aim of activities is achieving a good result (saves only "nice" drawings, for example)	m5_2	.000	.000	.033	.868 *	1.000
Receiving recognition for the final product/result is important	m5_3	.000	.000	.167	.868	.938
**Is able to work with others towards a common goal, e.g. in team sports and group activities**	m5_4	.000	.000	.000	.658 **	.938
**Participates in group activities of a competitive nature, such as races, bowling, etc.**	m5_5	.000	.000	.000	.605 ***	.938

*Note*. *N* = 160. Response probabilities of relevant age groups are highlighted in grey. The reference age group is shaded.

* *p* < .05.

** *p* < .01.

*** *p* < .001.

Appropriate items (response probability > 45%; age group specificity) are printed in bold letters.

**Table 8:** Response probability at item level; domain: Communication.

		Proportion of ‘yes’ responses per age group				
Content	Item	0–6 months	7–18 months	19–36 months	37–84 months	85–156 months
**Undirected expression of inner states using the entire body**	c1_1	.700	.067 ***	.000	.000	.000
**Verbal communication and comprehension are lacking**	c1_2	.767	.000 ***	.000	.000	.000
**Conveys emotional states by crying, shrieking, clinging to caregivers,** **pacing back and forth**	c1_3	1.000	.600 ***	.033	.000	.000
**Imitates facial expressions, sounds and words**	c1_4	.900	.567 **	.067	.000	.000
Keeps repeating the same words and sounds	c1_5	.667	.500	.067	.000	.000
Frequently clings to significant others	c2_1	.200 *	.433	.267	.000	.000
**Seeks constant physical and verbal contact with significant others by remaining close by their side**	c2_2	.100 ***	.633	.167 ***	.000	.000
Points at things to get others' attention	c2_3	.100 ***	.733	.600	.000	.000
Repeats sounds, words and short sentences he/she hears	c2_4	.200 ***	.833	.533 *	.000	.000
Uses his/her entire body to get his/her message across	c2_5	.300 ***	.967	.800	.000	.000
**Often says ‴no" to try to get his/her way**	c3_1	.000	.400 ***	.967	.316 ***	.094
**Communication is self-centered**	c3_2	.000	.100 ***	.733	.184 ***	.000
**Communication is mostly related to the immediate situation**	c3_3	.000	.167 ***	.600	.184 ***	.000
Uses "bad" words to provoke reactions from others	c3_4	.000	.000	.133	.316 *	.000
Says things with no regard for the social context	c3_5	.000	.000 ***	.333	.211	.000
**Often asks "why" questions**	c4_1	.000	.000	.167 ***	.816	.250 ***
**Asks questions and contributes information in conversations** **relating to his/her personal experience and daily life**	c4_2	.000	.000	.200 ***	.947	.625 ***
**Describes his/her emotions in simple terms**	c4_3	.000	.000	.433 ***	.974	.469 ***
**Can explain in simple terms why he/she is upset, happy, scared, jealous, proud etc.**	c4_4	.000	.000	.333 ***	.921	.469 ***
**Communication is directed at peers as well as significant others**	c4_5	.000	.000	.500 ***	1.000	.719 ***
**Asks questions about cause and effect and how things work**	c5_1	.000	.000	.033	.737 ***	1.000
Communicates with peers and enjoys exchanges with them	c5_2	.000	.000	.133	.921	1.000
Uses objective/factual arguments to support his/her opinion in discussions	c5_3	.000	.000	.067	.789 *	.938
**Is able to talk about his/her feelings**	c5_4	.000	.000	.067	.605 ***	1.000
**Is able to talk about his/her strengths and weaknesses**	c5_5	.000	.000	.033	.632 ***	.969

*Note*. *N* = 160. Response probabilities of relevant age groups are highlighted in grey. The reference age group is shaded.

* *p* < .05.

** *p* < .01.

*** *p* < .001.

Appropriate items (response probability > 45%; age group specificity) are printed in bold letters.

**Table 9:** Response probability at item level; domain: Regulating Affect.

		Proportion of ‘yes’ responses per age group				
Content	Item	0–6 months	7–18 months	19–36 months	37–84 months	85–156 months
**Tension is relieved by (active or passive) movement and physical touch**	a1_1	.900	.333 ***	.000	.000	.000
**Responds to physical sensations and external stimuli with tension**	a1_2	.933	.467 ***	.033	.000	.000
**Uses repetitive movements (e.g. rocking, flapping arms, hitting etc.) as a means of self-soothing**	a1_3	.300	.033 **	.033	.000	.000
**Has no control over his/her impulses**	a1_4	.833	.100 ***	.000	.000	.000
Displays auto-aggressive behavior	a1_5	.000	.033	.033	.000	.000
Negative emotions are regulated when the cause of stress is eliminated	a2_1	.800	.967	.733 *	.053	.031
Turns to significant others for protection and consolation	a2_2	.533 ***	1.000	.833 *	.132	.031
**Is able to control (aggressive) impulses if significant others** **are nearby and ready to intervene if necessary**	a2_3	.500 ***	.967	.567 ***	.026	.031
**Is upset and angry when significant others leave**	a2_4	.233 **	.567	.233 **	.026	.000
Frustration leads to anger that can be manifested in aggressive behavior towards significant others, physical restlessness, screaming, hitting or throwing objects	a2_5	.067 **	.433	.533	.053	.031
Responds with aggression when limits are imposed on his/her will	a3_1	.000	.500 *	.767	.474 *	.125
**Is more willing to cooperate when offered a choice between two alternatives**	a3_2	.000	.300 ***	.967	.553 ***	.062
**Frustration finds expression physical restlessness, temper tantrums** **and stubbornly oppositional behavior**	a3_3	.000	.200 ***	.900	.421 ***	.156
Frequently conveys protest using physical or verbal means	a3_4	.000	.300 *	.600	.289 **	.188
**Is rarely able to talk about the causes and effects of his/her aggressive behavior**	at3_5	.000	.033 ***	.800	.316 ***	.062
**Expresses regret and wants to make amends**	a4_1	.000	.000	.400 ***	.947	.719 **
Talks about what he/she regards as good or acceptable behavior and gives reasons	a4_2	.000	.000	.133 ***	.658	.688
Can delay gratification of his/her own wishes for the sake of a future reward	a4_3	.000	.000	.500 ***	.895	.719 *
**Is able to talk about the causes and effects of his/her aggressive behavior** **with encouragement from significant others**	a4_4	.000	.000	.200 ***	.868	.500 ***
Is a "sore loser" and refuses to continue playing after losing a game	a4_5	.000	.000	.033 ***	.474	.219 *
**Aggression is primarily expressed using verbal means**	a5_1	.000	.000	.167	.711 **	.969
**Is able to regulate his/her emotions by talking about his/her feelings and needs and seeking support from others in the group**	a5_2	.000	.000	.067	.447 **	.750
**Reflects on what did or didn't go well without prompting from others**	a5_3	.000	.000	.033	.711 **	.969
Responds with aggression when he/she feels threatened, slighted or extremely frustrated	a5_4	.000	.000	.100	.711	.875
Displays anger or aggression in competitive situations	a5_5	.000	.000	.033	.237	.312

*Note*. *N* = 160. Response probabilities of relevant age groups are highlighted in grey. The reference age group is shaded.

* *p* < .05.

** *p* < .01.

*** *p* < .001.

Appropriate items (response probability > 45%; age group specificity) are printed in bold letters.

In the following section, the item validity results are presented domain-wise for items considered as inappropriate: that is, items showing low response probabilities in the target age group or low levels of significance (>.01) compared to the adjacent age groups.

#### Relating to his/her own body

In this domain, most items met the criteria for appropriateness. Items b1_5, b2_3, and b3_5 occurred rarely in all age groups. Items b2_5, b4_3, and b5_2 indicated phase non-specificity, as the proportion of individuals in the reference group did not show a specific behaviour significantly more often than did individuals in adjacent age groups. However, in each level of ED, at least 3, but mostly 4 items met the designated criteria.

#### Relating to significant others

In phases 2 and 4, only two items each met the inclusion criteria. Items s1_5, s2_1, s2_3, s2_4, s4_1, s4_3, s4_5, s5_1, and s5_3 did not differ significantly from adjacent age groups in terms of the proportion of ‘yes’ answers. Item s3_4 showed low response probabilities in all age groups.

#### Dealing with change / object permanence

Only item o3_5 showed low response probabilities in the target age group. Two items in phase 2 (o2_4 and o2_5) and phase 3 (o3_1 and o3_2) were not sufficiently age-specific; neither were four items in phase 4 (o4_1, o4_2, o4_3, and 4_4) and three items in phase 5 (o5_1, o5_2, and o5_5).

#### Differentiating emotions

Items e1_3 and e1_4 showed low response probabilities in all age groups. In SED-2, no item met the level of significance for describing age-specific behaviour. For the same reason, six more items across different levels of ED were rated as inappropriate (e3_2, e3_4, e4_1, e4_4, e4_5, and e5_1).

#### Relating to peers

In this domain, only one to two items in each SED level met the criteria of appropriateness. Most items were not age-specific: i.e. items p1_2, p1_4, p2_2 to p2_5, p3_2, p3_3, p4_1, p4_4, p4_5 and p5_1 to p5_3, while three items described behaviour rarely seen in all children (p1_3, p1_5, and p3_5).

#### Engaging with the material world

In this domain, 18 of the overall 25 items met the inclusion criteria. Three items in phase 5 (m5_1 to m5_3) and one in each of the other levels of ED (m1_4, m2_2, m3_4 and m4_1) described behaviours that were not specific to the respective age group.

#### Communicating with others

Only one item in phase 2 (c2_2), but all items in phase 4, were deemed appropriate for the scale. In the other levels of ED, one to two items did not meet the necessities for specificity (c1_5, c5_2 and c5_3) or response-probability (c3_4 and c3_5).

#### Regulating affect

Two to four items described age-specific behaviours in each age group. Three items were reported in less than 45% of the target age group (a1_5, a2_5, a5_5), and another eight indicated phase-non-specificity (a2_1, a2_2, a3_1, a3_4, a4_2, a4_3, a4_5, a5_4).

### Criterion validity at domain level and overall agreement

**Fig 1:**
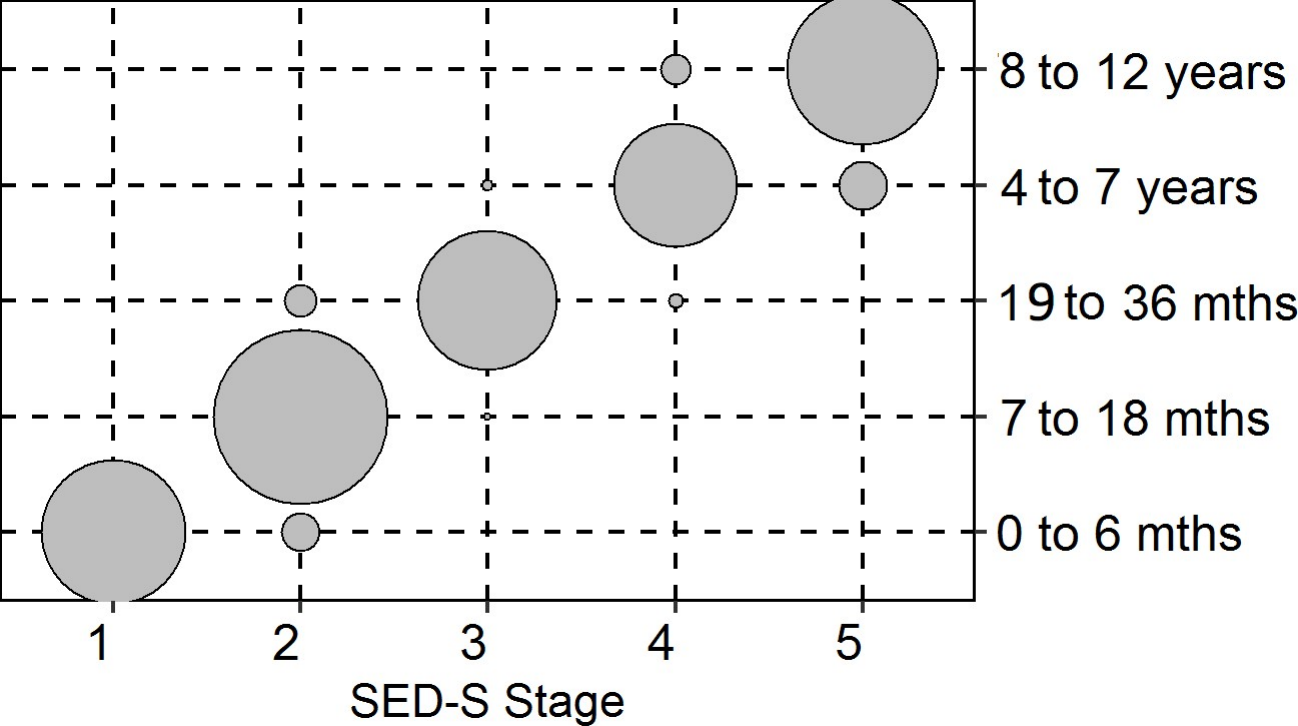
Agreement of the SED-S overall score and the respective age groups.

The agreement of the classifications of the eight SED-S domains with the chronological age group ranged between 65.6% (*Relating to Peers*, *κ*_*w*_ = 0.92) and 78.1% (*Relating to his/her Own Body*, *κ*_*w*_ = 0.94), *Mdn* = 70.9%. Kappa was 0.95 for the overall classification, with an exact agreement of 80.6%. Domain-wise rates of agreement, the corresponding kappa values, the proportions of individuals within a certain age group, and the classification in the SED-S phases are summarized at the domain level in Table 10 and at the scale level in Fig 1.

**Table 10:** Proportions of individuals within a certain age group, and the classification in the SED-S phases at the domain level.

Children aged	0–6 months	7–18 months	19–36 months	37–84 months	85–156 months	0–6 months	7–18 months	19–36 months	37–84 months	85–156 months	0–6 months	7–18 months	19–36 months	37–84 months	85–156 months
SED-S	Domain 1: Relating to his/her Own Body					Domain 2: Relating to Significant Others					Domain 3: Object Permanence				
	(κ = .94, 95% CI [.91, .96], exact = 78.1%)					(κ = .93, 95% CI [.91, .95], exact = 74.4%)					(κ = .91, 95% CI [.88, .94], exact = 66.9%)				
SED-S 1	.73	.00	.00	.00	.00	.73	.03	.00	.00	.00	.80	.10	.00	.00	.00
SED-S 2	.27	.90	.07	.00	.00	.27	.90	.13	.00	.00	.20	.83	.00	.00	.00
SED-S 3	.00	.10	.90	.18	.03	.00	.07	.83	.11	.00	.00	.07	.30	.03	.00
SED-S 4	.00	.00	.03	.58	.12	.00	.00	.03	.66	.38	.00	.00	.60	.45	.00
SED-S 5	.00	.00	.00	.24	.84	.00	.00	.00	.24	.62	.00	.00	.10	.53	1.00
	Domain 4: Differentiating Emotions					Domain 5: Relating to Peers					Domain 6: Engaging with the Material World				
	(κ = .93, 95% CI [.90, .95], exact = 72.5%)					(κ = .92, 95% CI [.89, .93], exact = 65.6%)					(κ = .93, 95% CI [.91, .95], exact = 71.9%)				
SED-S 1	.63	.00	.00	.00	.00	.67	.07	.00	.00	.00	.73	.03	.00	.00	.00
SED-S 2	.37	1.00	.30	.00	.00	.33	.83	.43	.00	.00	.27	.93	.10	.00	.00
SED-S 3	.00	.00	.67	.18	.00	.00	.10	.40	.05	.00	.00	.03	.70	.05	.00
SED-S 4	.00	.00	.03	.63	.28	.00	.00	.17	.55	.16	.00	.00	.20	.39	.09
SED-S 5	.00	.00	.00	.18	.72	.00	.00	.00	.39	.84	.00	.00	.00	.55	.91
	Domain 7: Communicating with Others					Domain 8: Regulating Affect					Overall score: SED-S overall				
	(κ = .93, 95% CI [.91, .95], exact = 69.4)					(κ = .91, 95% CI [.88, .94], exact = 70.0%)					(κ = .95, 95% CI [.93, .97], exact = 80.6%)				
SED-S 1	.93	.23	.00	.00	.00	.67	.03	.00	.00	.00	.80	.00	.00	.00	.00
SED-S 2	.07	.73	.43	.00	.00	.33	.93	.17	.00	.00	.20	.97	.17	.00	.00
SED-S 3	.00	.03	.40	.05	.00	.00	.03	.77	.24	.06	.00	.03	.77	.05	.00
SED-S 4	.00	.00	.17	.50	.06	.00	.00	.07	.53	.28	.00	.00	.07	.68	.16
SED-S 5	.00	.00	.00	.45	.94	.00	.00	.00	.24	.66	.00	.00	.00	.26	.84

*Note*. *N* = 160. The expected stage is highlighted in grey. Kappa-coefficients and exact agreement are shown at the domain level.

A summary of the valide items will be provided in the supplement.

### Sex differences

The analyses of the square weighted kappas for boys and girls separately on domain and overall SED-S level is shown in Table 11. No obvious sex differences could be found.

**Table 11:** Kappa-values and corresponding 95%-CI in the SED-S Domains and the overall score.

	**all**		**male**		**female**	
	**κ**	**[95% CI]**	**κ**	**[95% CI]**	**κ**	**[95% CI]**
Relating to his/her Own Body	.94	[.91, .96]	.95	[.91, .98]	.93	[.89, .95]
Relating to Significant Others	.93	[.91, .95]	.92	[.89, .95]	.94	[.91, .96]
Object Permanence	.91	[.88, .94]	.92	[.87, .95]	.91	[.86, .94]
Differentiating Emotions	.93	[.90, .95]	.93	[.90, .96]	.92	[.88, .95]
Relating to Peers	.92	[.89, .93]	.93	[.90, .96]	.90	[.86, .93]
Engaging with the Material World	.93	[.91, .95]	.93	[.90, .96]	.93	[.90, .96]
Communicating with Others	.93	[.91, .95]	.93	[.90, .96]	.93	[.89, .95]
Regulating Affect	.91	[.88, .94]	.90	[.85, .94]	.92	[.89, .95]
SED-S overall	.95	[.93, .97]	.95	[.93, .97]	.95	[.92, .97]

The comparison of the medians of boys and girls on domain and overall score level can be seen in Table 12.

**Table 12:** Medians of SED-S in the different domains and the overall score in boys and girls.

	Median SED-S Phase in Age Group 1			Median SED-S Phase in Age Group 2			Median SED-S Phase in Age Group 3			Median SED-S Phase in Age Group 4			Median SED-S Phase in Age Group 5		
	Male	Female	*p*	Male	Female	*p*	Male	Female	*p*	Male	Female	*p*	Male	Female	*p*
Age in month	**3**	**6**	**.047**	11	11	.420	29	25	.412	62	64	.828	126	121	.837
Relating to his/her Own Body	1	1	.185	2	2	.582	3	3	.775	4	4	.176	5	5	.955
Relating to Significant Others	**1**	**2**	**.047**	2	2	.328	3	3	.389	4	4	.460	4	5	.099
Object Permanence	1	1	.070	2	2	.171	4	4	.624	5	5	.696	5	5	1
Differentiating Emotions	1	1	.999	2	2	1	3	3	.267	4	4	1	5	5	1
Relating to Peers	**1**	**2**	**.030**	2	2	.350	3	3	.935	4	5	.196	5	5	.925
Engaging with the Material World	**1**	**2**	**.047**	2	2	1	3	3	.436	5	5	.613	5	5	.694
Communicating with Others	1	1	.420	2	2	.525	3	3	.567	5	4	.276	5	5	.512
Regulating Affect	**1**	**2**	**.030**	2	2	1	3	3	.775	4	4	.675	5	5	.377
SED-S overall	1	1	.070	2	2	.703	3	3	.775	4	4	.696	5	5	.639

Significant differences are marked in bold letters.

In the first age group (0 to 6 months), in 4 out of the 8 domains, girls showed significantly higher median values than the boys. In this age group, the girls were also significantly older than the boys (6 vs. 3 months).

### Internal consistency and within-profile homogeneity

The internal consistency was high, at α = .99. Overall, the majority of individuals provided homogenous profiles, with 33.8% of individuals showing the same phase across all domains and 53.8% showing a min-max difference of one, 11.3% showing a min-max difference of two and 1.3% showing a min-max difference of three SED levels.

### Inter-Rater reliability

Except for *Regulating Affect* and *Differentiating Emotions* (96% exact agreement, *κ*_*w*_ = .98), kappa coefficients for the agreement between the domain-wise and the overall classification were 1.00 (100% exact agreement).

## Discussion

The current study normed the SED-S, a scale for the assessment of ED, in a sample of typically developing children, and provides evidence for the criterion validity on item, domain and scale level. The majority of children showed homogenous profiles. Interrater reliability and internal consistency were high.

At item level, the expected pattern emerged for the majority of items: that is, the response probabilities were highest in the target age group and significantly higher compared to the adjacent age groups. Thus, the behaviours described in items which were considered as being ‘appropriate’ are typical for the assigned level of development. However, some items were observed in fewer than half of the children in the determined age group or were not sufficiently age-specific, and therefore should be excluded or rephrased in a revision of the SED-S.

At the domain level, quadratic weighted kappa-values were high and the exact agreement was good. However, the assignment to the assumed level of ED did not fit perfectly to the respective chronological age of the children. This may be caused not only by limitations of the instrument itself, but also by differences in the developmental stage of the various children. Also, in typically developing children, the day-to-day care and the living situation, including the presence and age of siblings, environmental factors and genetic aspects, may lead to more or less advanced levels of ED. Moreover, the age of some children was towards the lower or higher end of the respective age group, which increases the likelihood for assignment to an adjacent stage. The agreement between the children’s chronological age and the SED-S was best for the overall result of the SED-S. Assessment across several domains thus increased the overall accuracy of the scale.

No obvious sex differences could be found for the agreement of the child’s chronological age with the SED-S classification in terms of the quadratic weighted kappa values. Comparing the median values of boys and girls on domain and overall score level resulted in marginal differences in the first age group (0 to 6 months), with 4 out of the 8 domains showing significantly higher median values in girls than the boys. However, the girls were also significantly older than the boys (6 vs. 3 months). If the analysis is corrected for multiple testing (.05/8 = .006), the results are not significant any more. To conclude, we ascribe these marginal differences to the different ages of boys and girls within this age group.

Sex differences in emotional and social functioning are reported in various studies [26, 27]. Functional connectivity of the amygdala with other brain regions involved in stress and emotion processing seems to be sex-specific [28]. However, looking at our data did not reveal any apparent difference between the two groups. Maybe the size of the study sample is not large enough to detect sex specific differences.

Persons with intellectual disability show high prevalence rates of challenging behaviours [18]. So far, assessment of challenging behaviour focusses on environmental aspects: for example, behavioural analysis (NICE 2015). Due to the lack of appropriate concepts and measurements, intrinsic factors that are crucial for adaptive functioning, such as the level of mental functioning, remain under-investigated [7]. Despite the presence of different measures for emotional development, such as the *Scheme of Appraisal of Emotional Development* (SAED) [4, 29] the *Schaal voor het sociaal-emotionele ontwikkelingsniveau* (ESSEON-R) [30], the *Developmental–Structuralist Approach* (Greenspan; 1997a, b), the *Levels of Emotional Awareness* [31] or the *Infant-Toddler Social and Emotional Assessment scale* (ITSEA) [32], no study to date has normed the respective scale in a sample of typically developing children. Thus, the described characteristics and behaviours of the various models and measures have never been validated alongside the trajectories of typical development. The current study fills this gap and can be regarded as a proof of concept of the model using corresponding reference ages of typically developing children. Moreover, the above mentioned scales conceptualize ED less comprehensive and more focused on emotional aspects as such, while the SED-S comprises a thorough assessment of various aspects of mental functioning observable alongside the developmental trajectory of the different parts of the limbic system as described in the introduction. Finally, the SED-S is applicable to adults as the items have been phrased accordingly, while the above mentioned measures for ED are designed to be used in children. Each level of development is related to specific emotional needs, motivations and adaptive strategies and leads to certain observable behaviours. This may add a further perspective on the origins of challenging behaviours in persons with developmental delays and support practising psychiatrists in their diagnostic work-up.

In this study, internal consistency was high. In fact, a study by La Malfa et al. [33], which examined the psychometric properties of the SAED, showed similar values (Cronbach’s alpha: .96). Moreover, the proportion of infants showing a min-max-difference greater than 1 was small. This further supports the validity of the SED-S, as typically developing children should show rather homogenous profiles and therefore should be classified in the same or at least adjacent phases across all domains. To conclude, the different items seem to measure different elements of the same aspect, which in this case is ED.

The interrater reliability was very high, as indicated by high exact agreement and quadratic weighted kappa-coefficients above .95. This is comparable to the study conducted by La Malfa et al. (2009; kappa = .75) which assessed the psychometric properties of the SAED [33]. However, the estimate of interrater reliability in the current study may be increased by the fact that both raters participated in the same assessment and scored the same information. Therefore, interrater reliability must be reassessed by two independent evaluations of the raters.

Nonetheless, some limitations of this study have to be considered. The study popula-tion is a selected sample and may not represent the whole population of typically de-veloping infants. The development of the assessed infants was considered ‘typical’ based on the caregivers’ reports and a review of the child’s personal health record; the children were not re-examined by a physician from the study team. The raters were not blinded to the children’s chronological age. Cultural aspects were not addressed systematically. The interview was applied to only one parent, mostly the mothers, despite a multi-informant approach is considered as the best practice for assessment of emotional functioning in children [34]. In this study, Fält et al. (2018) reported a moderate agreement between mother and father ratings (ICC 0.66–0.76) with regard to the child’s problem behavior, while another study points out the unique information of mothers and fathers about their child’s behavioural and emotional problems [35]. Further studies may address the impact of the parents sex by comparing the results of interviews of mothers and fathers.

For the assessment of interrater reliability, both assessors rated the same structured interview. In a next step, the interview should be applied twice by two different raters to additionally assess the reliability of the application of the instrument. Moreover, the current estimates of inter-rater agreement are limited by the fact that only 25 individuals were investigated. Therefore, these results need to be replicated. We suggest using different approaches to inter-rater reliability in further studies, that is, testing comparing the coding of completely independent assessments and using a larger number of interviewers to get a better impression of the raters specificity and generalizability across different interviewers.

Finally, application and validation of the SED-S in an adult IDD population, with and without additional mental and behavioural disorders is pivotal to further validate the scale for its respective usage.

The SED-S showed adequate psychometric properties in terms of criterion-related validity and therefore may be applied in clinical practice to assess the level of ED. The application of the SED-S in a sample of adults with ID and an assessment of its psychometric properties would be the next step to ensure the empirical basis of the scale in this population. We therewith aim to provide a proof of concept for the assessment of the level of ED to add a further perspective on the basic emotional needs, self-regulation strategies and behaviours of persons with developmental delays.
